# Changes in Ion Selectivity Following the Asymmetrical Addition of Charge to the Selectivity Filter of Bacterial Sodium Channels

**DOI:** 10.3390/e22121390

**Published:** 2020-12-09

**Authors:** Olena A. Fedorenko, Igor A. Khovanov, Stephen K. Roberts, Carlo Guardiani

**Affiliations:** 1 Division of Biomedical and Life Sciences, Lancaster University, Lancaster LA1 4YE, UK; s.k.roberts@lancaster.ac.uk; 2 School of Engineering, University of Warwick, Coventry CV4 7AL, UK; i.khovanov@warwick.ac.uk; 3 Department of Physics, Lancaster University, Lancaster LA1 4YW, UK; carlo.guardiani@uniroma1.it

**Keywords:** ion channel, selectivity, permeability, patch-clamp, computer simulations

## Abstract

Voltage-gated sodium channels (NaVs) play fundamental roles in eukaryotes, but their exceptional size hinders their structural resolution. Bacterial NaVs are simplified homologues of their eukaryotic counterparts, but their use as models of eukaryotic Na^+^ channels is limited by their homotetrameric structure at odds with the asymmetric Selectivity Filter (SF) of eukaryotic NaVs. This work aims at mimicking the SF of eukaryotic NaVs by engineering radial asymmetry into the SF of bacterial channels. This goal was pursued with two approaches: the co-expression of different monomers of the NaChBac bacterial channel to induce the random assembly of heterotetramers, and the concatenation of four bacterial monomers to form a concatemer that can be targeted by site-specific mutagenesis. Patch-clamp measurements and Molecular Dynamics simulations showed that an additional gating charge in the SF leads to a significant increase in Na^+^ and a modest increase in the Ca^2+^ conductance in the NavMs concatemer in agreement with the behavior of the population of random heterotetramers with the highest proportion of channels with charge −5*e*. We thus showed that charge, despite being important, is not the only determinant of conduction and selectivity, and we created new tools extending the use of bacterial channels as models of eukaryotic counterparts.

## 1. Introduction

Voltage-gated sodium and calcium channels (NaVs and CaVs, respectively) are involved in a multitude of processes, including electrical signaling, secretion, and synaptic transmission [1]. The malfunction or dysregulation of NaVs and CaVs leads to a wide range of neurological, cardiovascular, and muscular disorders, including periodic paralysis [2], arrhythmia [3], and epilepsy [4], which highlights the importance of these molecules.

Eukaryotic NaVs and CaVs have similar structure and comprise a pore-forming α1 subunit of approximately 190–250 kDa, which co-assembles with a number of auxiliary subunits. The α1 subunit is organized in four domains, each comprising a voltage sensor (encompassing helices S1–S4) and a pore domain (including helices S5–S6) [5,6,7,8,9]. The four domains arranged around the pore are not identical, resulting in a channel structure that is asymmetric and pseudo-tetrameric [10]. The atomic level resolution of the structure of these molecules is essential to understand their structure-function relationships, but this task is particularly challenging for eukaryotic NaV channels, due to them being membrane integral proteins [11] and is exacerbated by their particularly large size. Consequently, to date only the structure of a single eukaryotic NaV has been resolved at the atomic level (3.8 Å), but the channel was not electrophysiologically characterized [12].

Bacterial NaVs and CaVs are simplified homologues of eukaryotic channels. They are homotetrameric channels formed by four identical monomers corresponding to the four domains of the α1 subunit of eukaryotic channels [13,14,15,16]. Their minimalist structure has enabled the determination of high-resolution atomistic structures, which has allowed extensive structure-functional characterization with respect to their cation selectivity, gating, and binding of anesthetics e.g., [17,18,19]. Although a complete understanding of selectivity has not been arrived at, the availability of high-resolution structures has provided a detailed understanding of the atomistic-level interaction of ions in the Selectivity Filter (SF). Despite this, there is still no widely accepted predictive model for cation selectivity, representing gaps in our knowledge of the molecular mechanism of ion permeation and selectivity. Mutation studies suggest the fixed charge (Q*_f_*) of the SF to be one of the major determinants of selectivity and permeation [15,20,21,22,23,24,25,26,27,28,29,30,31,32]. The Q*_f_* charge is at the core of many theoretical models attempting to explain the physical origins of cation selectivity in ion channels. For example, cation conduction has recently been modelled within the framework of Ionic Coulomb Blockade (ICB) [24,25], an electrostatic model with the aim of predicting Na^+^ and Ca^2+^ permeability based on knowing the actual Q*_f_* value of the SF.

Although prokaryotic and eukaryotic channels show the same general architecture along the axis of the pore (an outer vestibule and an inner water filled cavity separated by a narrow SF), the use of prokaryotic channels as models of their eukaryotic counterparts is limited by their lack of radial asymmetry. In the case of the homotetrameric bacterial NaVs, four identical monomers form the channel pore; in contrast, their eukaryotic counterparts are composed of four non-identical domains which introduce significant radial asymmetry [14,26,27,28,29,30,31]. This difference becomes evident at the level of the SF, where conduction and selectivity are controlled by a DEKA ring (Q*_f_* = −1*e*) in eukaryotic NaVs and an EEEE ring (Q*_f_* = −4*e*) in prokaryotic NaVs. Another puzzling fact is that the EEEE locus is typical of bacterial sodium-selective channels but also characterizes calcium-selective eukaryotic channels [16,32,33,34], thus leading to NaChBac being initially predicted to be Ca^2+^-selective. The existence of disparate sequences indicates that bacterial and eukaryotic channels enforce their ion preferences through different molecular strategies [15,34,35]. As a result, the selectivity and conduction mechanisms discovered in prokaryotes are not readily transferable to eukaryotes.

The puzzling functional similarity between bacterial NaVs and eukaryotic CaVs has been termed the “EEEE paradox” [36]. The paradox arises as a result of the violation of the assumption that Q*_f_* is the main driving force of cation selectivity. A possible resolution of the paradox is related to the existence of a conserved D residue in domain 2 of CaVs in the neighborhood of the EEEE ring. Monte Carlo simulations predicted this D residue (termed D2p51 in [37]) to occupy a position in close proximity to the EEEE locus. This observation led to the hypothesis that the locus imparting Ca^2+^ permeability is actually EEEED, with a Q*_f_* value of −5*e* [25,37]. Moreover, when this conserved D residue in domain 2 of Cav1.2 (referred to as D707 in [29]) was replaced with neutral residues, a striking reduction in Ca^2+^ binding to the SF was measured [29]. These results suggest D707 to be an important cation binding determinant of eukaryotic channels.

The different behavior of prokaryotic and eukaryotic voltage-gated sodium and calcium channels highlights the importance of incorporating radial asymmetry in the SF of prokaryotic channels. In our previous work [38] we reported the creation of a concatenated bacterial NaV, in which four NavMs monomers were covalently linked to form a stable single polypeptide chain, resembling the general structure of a eukaryotic NaV. This allowed the targeted mutagenesis of individual domains introducing radial asymmetry in the bacterial channel with the aim to gain further insight on the role of Q*_f_* as a determinant of ion selectivity. In the present study, we report the first attempt to mutate the concatemer and generate a bacterial sodium channel with radial asymmetry in the SF. In order to obtain atomistic-level detail of selectivity and permeation, the electrophysiological characterization was integrated with Molecular Dynamics (MD) simulations of wild-type NavMs (Q*_f_* = −4*e*) and a mutant with an additional negative charge in the SF (Q*_f_* = −5*e*).

In the present study, we have also employed an independent yet complementary approach to introduce radial asymmetry into the SF of a bacterial sodium channel. Namely, a number of combinations of NaChBac monomers (differing in their amino acid composition and Q*_f_* value of the SF) were transfected into Chinese Hamster Ovary (CHO) cells to generate a random population of heterotetrameric channels with radial asymmetry in the SF. The mixed monomer approach using NaChBac monomers showed that Ca^2+^ conduction is increased in channels with a Q*_f_* > −4*e* (consistent with the proposed explanation for the EEEE paradox). Our data confirm the key role of the SF charge as the major determinant of conduction and selectivity. However, the failure to completely overturn the sodium selectivity of the NavMs concatemer to Ca^2+^ selectivity (with much smaller relative Ca^2+^ permeability exhibited by the −5*e* mutant NavMs concatemers compared to that for eukaryotic CaVs) suggests the existence of fine-tuning mechanisms of structural origin.

## 2. Materials and Methods

### 2.1. Materials Generation of Mutant Bacterial Channels

cDNA constructs encoding NaChBac (GenBank accession number BAB05220) and NavMs (GenBank accession number WP_011712479) bacterial sodium channels were synthesized by EPOCH Life Science (www.epochlifescience.com). NavMs concatemer was subcloned into pTRACER-CMV2 (Invitrogen) downstream of CMV promoter, as described previously [38].

Site-directed mutagenesis was performed using specific primers containing the sequence for the desired amino acid substitutions (according to Q5^®^ Site-Directed Mutagenesis Kit; New England BioLabs Inc., Hitchin, UK). For the generation of LEDWAS mutant from wild-type NaChBac, we used the forward primer CACGCTAGAGgatTGGGCGAGCG and the reversed primer ACCACTTGGAACAATGTTAAC; for LASWAS mutant, we used the forward primer GGTCACGCTAgccTCATGGGCGAGcggc and the reversed primer ACTTGGAACAATGTTAACAAACtaagc.

Q*_f_* = −5*e* NavMs mutants were generated from NavMs concatemer, which was designed with restriction sites delimiting each domain (Supplementary Figure S3A). Domain I (KpnI/EcoRI) and Domain II (EcoRI/EcoRV) were excised by restriction digest. The domain fragments were re-amplified by PCR using primer pairs to regenerate the restriction site prior to subcloning into vector pCR Blunt II-TOPO (Invitrogen): primers are Kpn1_NavMs_F (CCCGGTACCAGCCGCCACCATGTCACGCAAAATAAG)/EcoRI_ NavMs_R (CCCGAATTCGGGCTCGTCCTCCCAGATG) for Domain I and EcoRI_ NavMs_F (CCCGAATTCATGTCTAGGAAGATCC)/EcoRV_ NavMs_F (CCCGATATCGGGCTCGTCCTCCCAGATG) for Domain II. Site-directed mutagenesis (for S179D, according to NavMs monomer residue nomenclature) was performed on each domain using primers LEDWSM_ NavMs_F (GACCTTAGAGgatTGGTCTATGGGC) and LEDWSM_ NavMs_R (ATCACCTGAAATAGTGTG) prior to the restriction enzyme-mediated excision and ligation (T4 DNA ligase; NEB) of the Domain DNA fragment in the NavMs concatemer at sites KpnI/EcoRI (for Domain I) and EcoRI/EcoRV (Domain II).

All the clones were sequenced to check for correct construction and to ensure that no unwanted PCR induced mutations had been introduced. DNA for the transfection of cells was prepared using Midi Plasmid Kit (Qiagen, Manchester, UK).

### 2.2. Cell Culture and Transfection

Chinese hamster ovary (CHO) and human embryonic kidney (HEK293T) cell lines were obtained from Dr. Stephen K. Roberts. Cells were cultured in DMEM high glucose with L-glutamine (Lonza, Slough, UK ) supplemented with 10% Fetal Bovine Serum (Thermo Scientific, Loughborough, UK) with the addition of 50 U/mL of penicillin and 50 μg/mL of streptomycin (Sigma, Irvine, UK). Cells were maintained in a T25 flask (Thermo Scientific) at 37 ˚C in a 5% CO_2_ incubator and passaged twice a week. Then, 24 h before transfection, the cells were seeded in 6-well plates (Corning, Deeside, UK) containing No.1 coverslips (Scientific Laboratory Supplies, Nottingham, UK). A total of 10 μL of transfection reagent (Mirus, Cardiff, UK) and 5 μg of plasmid DNA or a mixture of DNAs in defined proportions were equilibrated separately in 250 μL of UltraMEM™ Reduced Serum Medium (Lonza, Slough, UK) at room temperature for 5 min and then mixed and incubated at room temperature for 20 min to form the DNA-reagent complex. Treated cells (at 80% confluency) were supplemented with DNA-reagent complex and incubated at 37 ˚C and 5% CO_2_ for 24–48 h before experiments.

### 2.3. Electrophysiology

Whole-cell voltage clamp recordings were performed at room temperature (20 ˚C) using an Axopatch 200A (Molecular Devices, Inc., Wokingham, UK) amplifier. Patch-clamp pipettes were pulled from borosilicate glass (Kimax, Kimble Company, Dover, USA) to resistances of 2–3 MOhm. Shanks of the pipette’s tip were coated with bee’s wax to reduce the pipette capacitance. The pipette solution contained (in mM) 15 Na-gluconate, 5 NaCl, 90 NMDG, 10 EGTA, and 20 HEPES, pH 7.4 adjustedwith 3 mM HCl). To record Na^+^ influx currents the bath solution was (in mM) 140 Na-methanesulfonate, 5 CsCl, 10 HEPES and 10 glucose (pH 7.4 adjusted with 4.8 mM CsOH); for the measurement of the Ca^2+^ influx currents, 140 mM of Na-methanesulfonate was replaced with 100 mM of Ca-methanesulfonate.

Data collection was initiated 3 min after obtaining the whole cell configuration to ensure the complete equilibration of the pipette solution and cytosol. The bath solution was grounded using a 3 M KCl agar bridge; the liquid junction potential determined experimentally (as described by [39]) agreed with that calculated (using JPCalc program, Clampex, Axon Instruments, Inc., Wokingham, UK) and was negligible. To ensure the complete exchange of the bath solution, electrophysiological recordings were initiated after >4 min of solution change. The rate of the gravity-fed perfusion system for the bath solution exchange was approximately 0.7 mL/min in a chamber volume of approximately 200 μL.

The results were analyzed using the Clampfit 10.1 software (Molecular Devices, Wokingham, UK) and OriginPro8 (OriginLab Corporation, Wellesley, MA, USA). Pooled data are presented as means ± SEM (*n*), where *n* is the number of independent experiments.

### 2.4. Equilibrium Simulations of NavMs Channel

The initial structure of wild-type NavMs was taken from the Protein Data Bank (PDB ID: 3ZJZ). Mutation S179D on chain A, and embedding in a membrane of 248 POPC molecules was performed using the CHARMM membrane builder [40,41]. The membrane was bathed on both sides by a 0.14 M NaCl solution or a 0.1 M CaCl_2_ solution. The size of the simulation box was 102 × 102 × 86 Å and the total number of atoms in the four simulated systems was a little short of 90,000. All the acidic residues have been assigned a charge −1*e*, while basic residues have been assigned a charge +1*e* based on an analysis of the pKa values with the PROPKA program (server.poissonboltzmann.org/pdb2pqr). All the simulations were performed with the NAMD 2.11b2 [42] suite of programs using the ff14SB [43] force field for the protein and the Lipid14 force-field [44] for the phospholipids. As already observed in [45], in the absence of harmonic restraints the pore rapidly closes at the cytoplasmic gate. In order to avoid this behavior that likely results from the absence of the Voltage Sensor Domain in the simulated system, harmonic restraints (50 kcal/mol/Å^2^) were applied to the backbone atoms of the transmembrane helices (residues 131–154 and 194–222) throughout the simulation. The four systems first underwent 10,000 steps of conjugate gradient minimization.

During equilibration, harmonic restraints were applied to non-hydrogen atoms of the protein backbone and side-chains (outside the transmembrane helices; residues 155–193), as well as to the phospholipid heads. A harmonic restraint was also applied to the dihedral angle formed by carbons 8, 9, 10, 11 of oleoyl acid and to the improper dihedral C1–C3–C2–O2 involving the three carbons of the glycerol unit and the hydroxyl oxygen linked to its central carbon. The equilibration was organized in six stages, whereby the constraints were gradually released. The values of the force constants used in the six stages can be found in Supplementary Table S1. The production run was carried out in the isothermal isobaric (NPT) ensemble for 100 ns (in NaCl solution) or 150 ns (in CaCl_2_ solution). The pressure was kept at 1 atm by the Nose–Hoover Langevin piston method, while the temperature was kept at 300 K by coupling to a Langevin thermostat with a damping coefficient of 1 ps^−1^. Long-range electrostatic interactions were evaluated with the smooth particle mesh Ewald algorithm. For the short-range non-bonded interactions, we used a cutoff of 12 Å with a switching function at 10.0 Å. The integration time step was 2 fs, and the bonds between hydrogen and heavy atoms were fixed to eliminate the most rapid oscillatory motions. The Potential of Mean Force (PMF) was computed using equation $F(z)=-k_{B}T\log(\rho (z)/\rho_{b})$, where $k_{B}$ is the Boltzmann constant, T is the absolute temperature, ρ(z) is the density profile of sodium or calcium ions, and $\rho_{b}$ is the density of these ions in the bulk. Since the ion density in the channel is typically higher than in the bulk, the PMF normally has negative values. To avoid a divergence in the logarithmic expression of the PMF, we assigned F(z)=0 when ρ(z)=0—that is, in the regions of the channel that are never visited by ions.

### 2.5. Current-Voltage Curves Calculation

Current–voltage (IV) curves in NavMs were attained using the collective diffusion model introduced in [46], where the time-course Q(t) of the net charge transported across the channel at equilibrium is thought of as an unbiased random walk. The net charge transported in the time interval Δt between two consecutive frames of the trajectory is $\Delta Q=\sum_{z_{1}\leq z\leq z_{2}}\frac{e_{i}\Delta z_{i}}{L_{z}}$, where the sum runs over all ions i, such that $z_{1}\leq z\leq z_{2}$, $z_{1}=-4.5$ Å and $z_{2}=16.5$ Å are the axial limits of the filter region somewhat extended in the vestibule and central cavity. The use of this extended SF gives us the opportunity to exploit the fluctuations due to ions exploring the vestibule region without entering into the SF as well as the aborted permeation events where the ion crosses the mouth of the SF but is immediately pulled back in due to the attraction of the acidic residues. In the expression, $\Delta z_{i}$ is the axial displacement of the ion in the time interval Δt and $L_{z}=z_{2}-z_{1}$ is the length of the SF. The time course of the charge, Q(t), can then be attained as $Q(t)=\sum_{t_{i}<t}\Delta Q(t_{i})$.

Diffusion theory predicts that, for sufficiently long times, the mean square displacement of the charge $\langle Q^{2}(t)\rangle$ grows linearly with a slope proportional to the diffusion coefficient, $\langle Q^{2}(t)\rangle \sim 2D_{Q}t+Const$. Applying linear response theory, the steady current induced by a small constant voltage V can be computed as $I_{steady}=D_{Q}V/k_{B}T$. Using such an approach, the linear region of an IV curve can be computed based on the spontaneous ion fluctuations at equilibrium in the absence of any applied electric field.

## 3. Results

### 3.1. Experimental Results

To introduce radial asymmetry in the SF of NaChBac, two approaches were adopted. First, mixed populations of NaChBac monomers (differing in their amino acid composition and the Q*_f_* value of the SF) were co-transfected into CHO cells to generate hetero-tetrameric channels exhibiting radial asymmetry in the SFs. Second, we used a concatenated NavMs tetramer [38] to generate radial asymmetry in the SF by the targeted mutation of one of the four repeats.

#### 3.1.1. Na^+^/Ca^2+^ Selectivity for Randomly Mixed Populations of NaChBac Monomers

The random assembly of channel tetramers can be demonstrated taking advantage of the different electrophysiological properties of WT NaChBac and the L226P mutant illustrated by the recordings in Supplementary Figure S1. The L226P mutation causes conspicuous alterations in channel gating of NaChBac from depolarization-activated whole-cell currents to non-inactivating hyperpolarization-activated whole-cell currents [47], (Supplementary Figure S1A,B). The mutation shifts the voltage of the maximal current from −10 mV in WT to −180 mV in the mutant (Supplementary Figure S1D), thus currents at −10 mV originating from separate channel populations of WT and L226P homotetramers can be easily separated. The current recordings from CHO cells co-transfected with NaChBac-encoding WT:L226P cDNAs in a ratio 3:1 (Supplementary Figure S1C,E) exhibited unique currents at −10 mV, which cannot be explained by the simple addition of whole current traces from homotetramer channels formed from either L226P or wild-type NaChBac (note that there is no current at −10 mV from L226P channels), indicating that unique heterotetramers are being formed. Assuming that the assembly of heterotetramers is formed without bias, the proportions of channel types can be determined by binomial distribution. It is noteworthy that this assumption is in agreement with previous findings [47,48], showing no bias for heterotetramer formation in CHO cells expressing a mixture of WT and G219P mutant NaChBac monomers and dimers.

**Figure 1:**
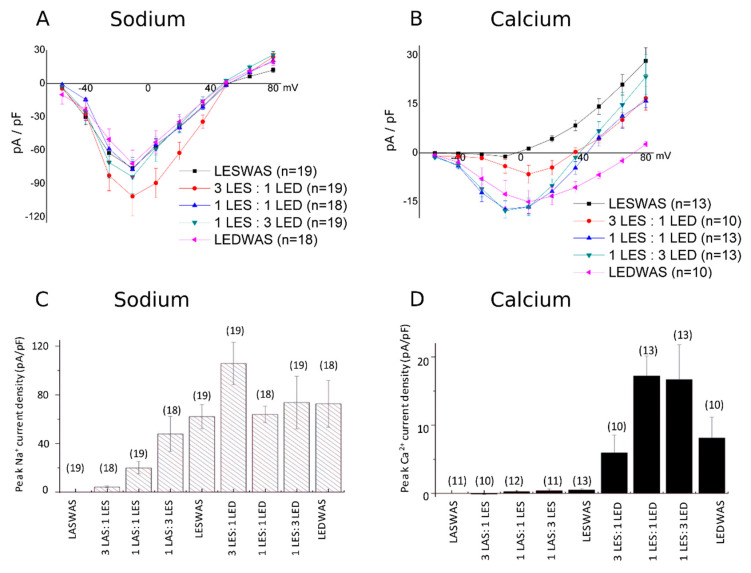
Na^+^/Ca^2+^ selectivity for NaChBac monomer mixtures. The voltage–current relations (**A**,**B**) and the mean (+/− SEM) whole-cell peak current density at −10 mV (**C**,**D**) for Na^+^ (**A**,**C**) and Ca^2+^ (**B**,**D**) in CHO cells transfected with cDNAs encoding for NaChBac channels possessing either a wild-type selectivity filter (LESWAS/LES) or a mutated selectivity filter (LASWAS/LAS or LEDWAS/LED); 5 µg of total DNA was used per transfection and was composed of either a mixture of types of cDNA at defined ratios, as indicated in Table 1 and on the *X*-axis, or a single cDNA type. Numbers in parentheses indicate the number of replicates.

Using this approach, CHO cells were co-transfected with cDNAs of NaChBac-encoding WT and mutants, with varied Q*_f_* in the SF, in different ratios. Note that the open probabilities and single channel conductances for the WT NaChBac (LESWAS) and LEDWAS homotetramers were equivalent (Supplementary Figure S2), and that the whole-cell Na^+^ currents from cells expressing homotetramer WT and LEDWAS were similar in magnitude (Figure 1a,c), consistent with the expression of the channel (i.e., number of channels) being independent of the single amino acid mutations introduced into the SF. Figure 1 shows the currents recorded from cells transfected with defined mixtures of NaChBac monomers; see Table 1 for the probabilities of different charged species, assuming that the assembly follows a binomial distribution.

Whole-cell currents were initially recorded in bath solution containing 140 mM of Na-methanesulfonate, followed by recordings after the complete replacement of bath Na^+^ with 100 mM of Ca-methanesulfonate. Na^+^ permeation appears relatively insensitive to changes in Q*_f_* values between −4*e* and −8*e* and equivalent in cells expressing only LESWAS and/or LEDWAS monomers. Focusing on channels exhibiting Q*_f_* values less than −4*e*, it is interesting to note that, despite co-transfection with a 1:1 ratio of LASWAS:LESWAS resulting in an expected only 6.2% of the channel population being homotetramers of LESWAS (Q*_f_* = −4*e*), the Na^+^ current density was approximately 30% of that recorded from cells expressing only LESWAS homotetramer channels (Figure 1a,c). An equivalent interpretation can be made for measurements of current density from cells transfected with a 1:3 ratio of cDNAs encoding LASWAS:LESWAS: the sodium current density was equivalent to that recorded from cells expressing only LESWAS homotetramers, despite only 32% of the channel population being predicted to be homotetrameric LESWAS. It is also interesting to compare the current density of the 3:1 LASWAS:LESWAS-expressing cells. Note that these cells show about 25% current density compared to the LESWAS-only cells (5 and 20 pA/pF, respectively). If one looks at the binominal predictions, 25% of channels are predicted to have Q*_f_* = −2*e* and greater and this is consistent with a Q*_f_* = −1*e* and 0 being non-conducting (Figure 1c). The simplest explanation for the disproportionately large Na^+^ current in cells expressing mixtures of LESWAS and LASWAS monomers is that functional NaChBac channels possessing a SF with a Q*_f_* value less than −4*e* are functional and able to mediate the Na^+^ influx.

Extending this type of analysis to the Ca^2+^ currents, cells transfected with a 1:3 ratio of LESWAS:LEDWAS encoding cDNAs (in which 0.3% of expressed functional channels were predicted to be LEDWAS homotetramers, with a Q*_f_* = −8*e*) exhibited a similar current density for Ca^2+^ influx as that from cells expressing only LEDWAS channels (Figure 1d). Thus, functional NaChBac channels possessing SFs with a Q*_f_* value of less than −8*e* appear to be able to mediate Ca^2+^ influx, with the possibility that a Q*_f_* value of −5*e* is sufficient to permit Ca^2+^ permeation. This explanation is also consistent with the observation that the Ca^2+^ current density is greatest in cells transfected with equal and 1:3 ratios of LESWAS:LEDWAS (Figure 1b,d). Note that the Na^+^ influx current density remains relatively constant in cells transfected with both LESWAS and LEDWAS encoding cDNAs, indicating that the effect of varying Q*_f_* between −4*e* and −8*e* was specific to the Ca^2+^ current density.

#### 3.1.2. Na^+^/Ca^2+^ Selectivity for Concatenated NavMS Channels

Although the use of a mixed population of cDNAs encoding for NaChBac and its mutants suggested the value of Q*_f_* to be a major determining factor for Na^+^/Ca^2+^ selectivity, the results are subject to the caveat that the whole-cell currents result from the cumulative current from an unknown but predictable range of different channel types. To address this complication, we attempted to generate a stable concatenation of NaChBac to enable the expression of a homogeneous population of NaChBac mutants; however, we have previously shown [38] the NaChBac oligomer to be unstable and not to remain intact in the plasma membrane. In contrast, an equivalent intact NavMs oligomer could be stably expressed in HEK293T cells [38] and thus enable the generation of a homogeneous population of bacterial channels, in which the Q*_f_* value of the SF can be altered in steps of 1*e*. The SF of eukaryotic CaVs is formed by a ring of glutamates (the EEEE locus) and a conserved aspartate residue in domain II (D2p51 [37]). The D2p51 residue is suggested to form a binding site for a third incoming Ca^2+^ from the extracellular side of the pore and thus bring an additional positive charge to the SF region necessary for the release of a bound Ca^2+^ to the cytosolic side (i.e., a knock-on mechanism [49]). Although direct evidence for the role of the D2p51 in Ca^2+^ permeation remains elusive, replacing the D2p51 residue in Cav1.2 (aka D707) with a neutral amino acid residue significantly reduces the Ca^2+^ binding of the SF [29]. Thus, to gain further insight into the role of the D2p51 in Ca^2+^ permeation, we used site-directed mutagenesis targeted to repeat I or II in the NavMs oligomer to generate a bacterial NaV with an “EEEED” locus (Q*_f_* = −5*e*) in the SF (Supplementary Figure S3). NavMs has a high homology (45% sequence identity) to NaChBac [28,45], which should enable comparison with the results from NaChBac.

**Figure 2:**
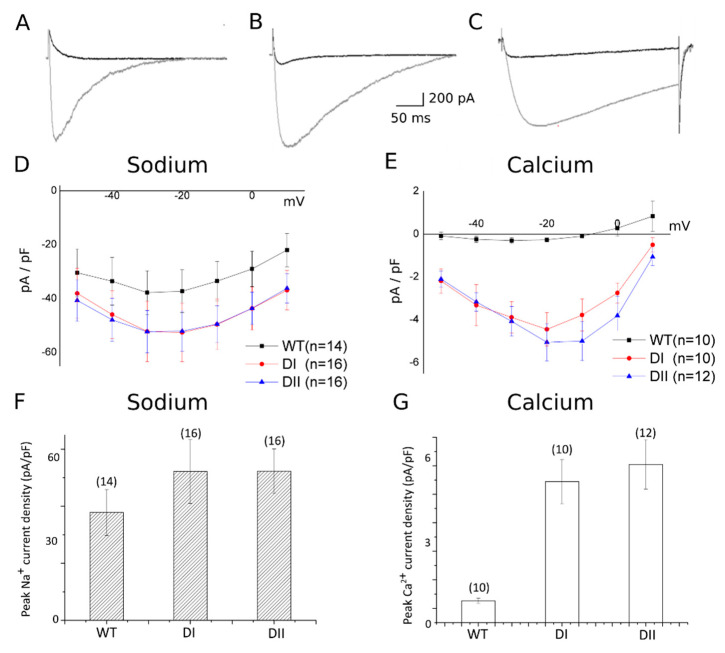
Na^+^/Ca^2+^ selectivity for NavMs concatemer possessing varied Q*_f_* values in their SF. The original recordings representatives of wild-type NavMS (**A**) and its DI (**B**) and DII (**C**) mutants in 140 mM Na^+^ solution (grey traces) and in 100 mM Ca^2+^ solution (black traces). The voltage–current relations (**D**,**E**) and the mean (+/− SEM) whole-cell peak current density at −10 mV (**F,G**) for Na^+^ (**D,F**) and Ca^2+^ (**E,G**) in HEK 293T cells transfected with cDNAs encoding for either wild-type or mutated NavMS. Numbers in parentheses indicate the number of replicates; and in HEK293T cells transfected with wild-type NavMS concatemer (WT) and mutant NavMS concatemer (DI and DII).

The WT NavMs SF is defined by ^177^LESWSM^182^, and we generated NavMs tetramers (Supplementary Figure S3) with the S179D mutation in either repeat I (mutant DI) or repeat II (mutant DII). Both mutants are therefore expected to carry a charge of −5*e* in the SF. Figure 2 shows typical whole-cell currents from WT and mutant NavMs in bath solution containing either 140 mM of Na^+^ or 100 mM of Ca^2+^.

In order to make quantitative comparisons between the electrophysiological behavior of NaChBac and NavMs mutants, the peak calcium and sodium currents as well as their ratio are tabulated in Supplementary Table S3 for the NaChBac heterotetramer populations and in Supplementary Table S2 for the NavMs mutants. The comparison of the data of the two tables shows that the ratio of the peak current densities for Na^+^ and Ca^2+^ in wild-type NavMs (0.018) is comparable to that for wild-type NaChBac (0.010). The tables also show that the ratio of peak current densities for Ca^2+^ and Na^+^ in the two NavMs mutants with an SF charge of −5*e* (0.080 for the DI mutant and 0.097 for the DII mutant) is similar to that for mutant channels formed from the expression of the 3LES:1LED mixture of NaChBac (0.054) in CHO cells, which yields the highest probability of occurrence (42%) of heterotetramers with an SF charge equal to −5*e*. Although both data sets support the increased Ca^2+^ permeability in −5*e* mutant bacterial sodium channels, the difference in the Ca^2+^ current magnitude that is evident upon comparing NavMs and NaChBac channels clearly indicates that factors other than the value of Q*_f_* are important in determining the Ca^2+^ permeability.

### 3.2. Computational Results

In an attempt to gain a molecular-level understanding of the different behavior of WT NavMs (Q*_f_* = −4*e*) and its mutant with charge Q*_f_* = −5*e*, we ran equilibrium MD simulations in a 100 mM solution of CaCl_2_ or 140 mM NaCl (for 150 and 100 ns, respectively). The initial structure of WT NavMs was taken from the Protein Data Bank (ID: 3ZJZ). Mutation S179D on chain A and embedding in a membrane of 248 POPC molecules was performed using the CHARMM membrane builder [40,41].

**Figure 3:**
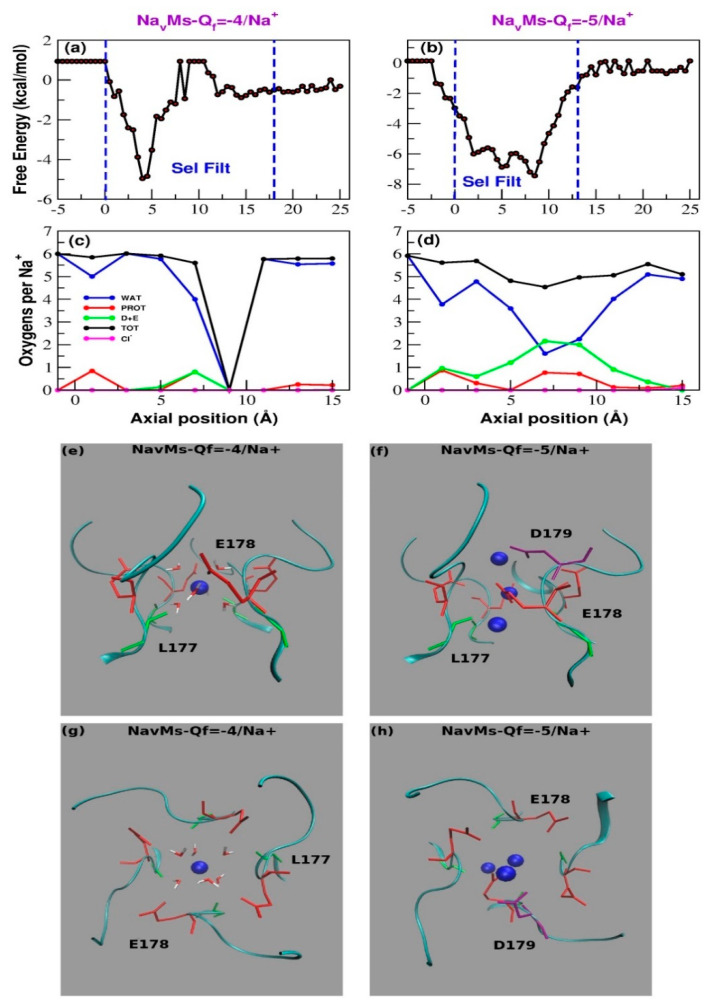
MD simulations for the WT and mutant NavMS in NaCl 140 mM. (**a**,**b**) Potential of Mean Force of Na^+^ as a function of the axial position in WT NavMS (**a**) and the mutant with charge Q*_f_* = −5*e* (**b**). (**c**,**d**) Average number of coordinating oxygens per sodium ion in axial bins with a thickness of 2.0 Å. (**c**) Wild-type NavMs; (**d**) NavMs mutant with Q*_f_* = −5*e*. The distance cutoff to identify sodium-chloride interactions was set to 3.5 Å, and for sodium-oxygen to 3.2 Å. Color code is as follows. Blue line: number of coordinating water-provided oxygens; green line: number of coordinating oxygens provided by aspartate and glutamates; red line; number of coordinating oxygens provided by other protein residues; black line: total number of coordinating oxygens; magenta line: number of coordinating chlorides. (**e**,**h**) Configuration of the selectivity filter of wild-type NavMS (**e**,**g**) and the mutant with charge Q*_f_* = −5*e* (**f**,**h**). All the structures correspond to the last frame of a 100 ns simulation in 0.14 M NaCl. Panels (**e**,**f**) show a side view of the SF; panels (**g**,**h**) show the top view. Glu178 is shown in red, while Asp179 is shown in purple. The backbone of Leu177 is shown in green. Sodium ions are portrayed as blue beads. Panels (**e**,**g**) also show the water molecules that mediate the interactions between the resident sodium ion and the protein in wild-type NavMS.

In 140 mM of NaCl solution, the WT NavMs SF is stably occupied by a single Na^+^ even if transient events of occupation by a second ion can also be spotted (Supplementary Figure S4a). Q*_f_* = −5*e* mutants SF is almost immediately occupied by two Na^+^ ions and, after 40 ns, the filter becomes stably occupied by three sodium ions (Supplementary Figure S4b). The different behavior of the two species is also reflected in the PMF profile (Figure 3a,b), which is characterized by a single deep minimum centered at z = 4–5 Å for WT NavMs, and a minimum split into three sub-basins at z = 2.0 Å, z = 5.0 Å, and z = 8–9 Å, corresponding to three different binding regions, for the Q*_f_* = −5*e* mutant. The barriers between the sub-basins are in the order of 1–2 kcal/mol and can be easily overcome at the simulation temperature, yet the sodium ions linger in each binding site for longer than they would in case of a uniform probability distribution of occupancy.

The nature of these binding sites can be better characterized by analyzing the conformation of the SF in the last frame of the 100 ns simulations (Figure 3e–h). A notable feature of wild-type NavMs is that the side chains of E178 residues do not point toward the center of the channel, but they are aligned along the channel wall pointing towards the extracellular side. As a result, the distance between the resident sodium ion and the ε-oxygen of E178 always exceeds 4.0 Å. This means that there are no direct sodium-protein interactions; Na^+^ interacts with the protein via water molecules in its hydration shell. Indeed, the withdrawn placement of E178 side chains leaves sufficient space in the SF for Na^+^ to fully keep its first hydration shell of six water molecules. In contrast, the conformation of the SF of Q*_f_* = −5*e* mutant revealed three sodium ions that directly interact with the residues of the SF; specifically, the extracellular one interacting with D179 and E178 both located on chain A, the central one with E178, and the intracellular sodium with the backbone carbonyl group of one of the L177 residues. The additional negative charge thus determines an enhanced ability of the NavMs mutant to capture sodium ions from the bulk. This, combined with the possibility of a knock-on mechanism deriving from the simultaneous presence of three Na^+^ ions in the SF, possibly explains the larger sodium current density for NavMs channels with Q*_f_* = −5*e*. As a result of this structural arrangement (and in contrast to that for the WT; Figure 3c), Na^+^ ions accessing the SF of the mutant lose on average 3.5 water molecules. However, this loss is compensated by the interactions with the oxygens provided by the acidic residues (2 oxygens) and by other protein residues (1 oxygen), such that the total number of coordinating oxygens is maintained equivalent to that for sodium in bulk solution (Figure 3c,d). Note that the interactions of the resident ions with all other residues of the SF are water-mediated.

In 100 mM of CaCl_2_ within the timescale of our simulations, no Ca^2+^ gains access to the SF of the WT channel, while a single Ca^2+^ enters into the SF of the Q*_f_* = −5*e* mutant during the early stages of the simulation and thereafter remains locked inside, while also repelling other potentially incoming calcium ions (Supplementary Figure S5a,b). This pattern is in keeping not only with the behavior of WT and mutant NavMs concatemer, but also with the results of the experiments on mixed populations of NaChBac heterotetramers. In fact, while the calcium peak current of the LESWAS homotetramers (Q*_f_* = −4*e*) is just 0.66 pA/pF, that of the 3LES:1LED population, where we expect the highest proportion of channels with Q*_f_* = −5*e*, is tenfold higher (6.0 pA/pF). The seeming mismatch between the currents recorded in experiments and the total block of the Ca^2+^ ion revealed by the simulations is (at least in part) due to the fact that, in the latter, no electric field was applied. Moreover, experimental recordings are performed on a timescale of hundreds of milliseconds, one million times longer than that covered by simulations, allowing time for slow, activated events of ion permeation. A comparison between our computational results and those by other groups is discussed in Supplementary Figure S6.

**Figure 4:**
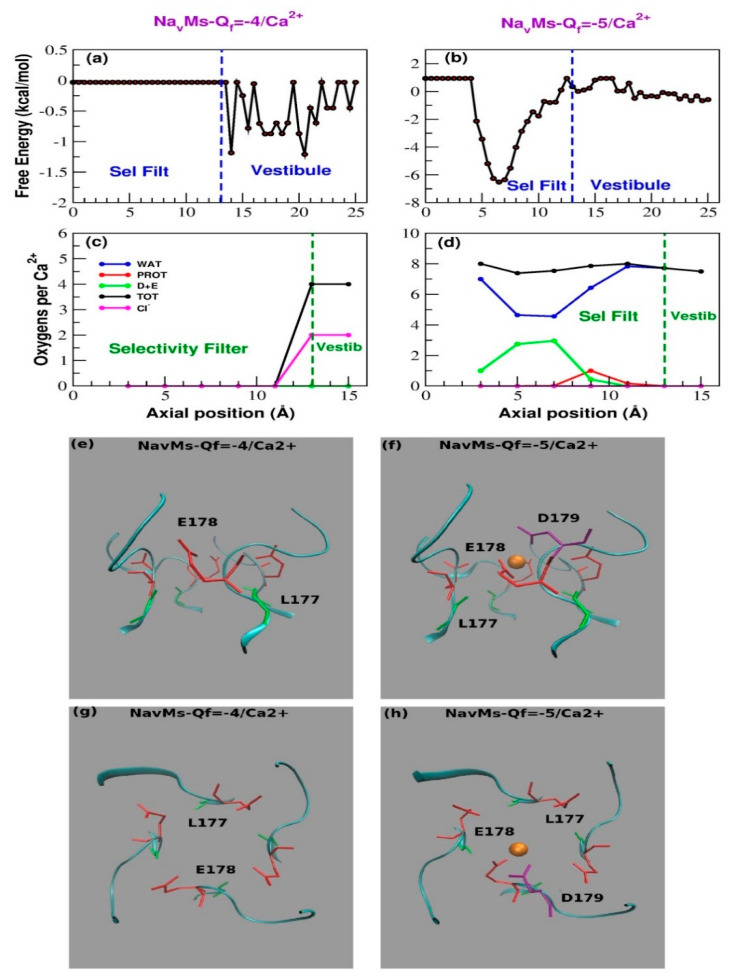
MD simulations for WT and mutant NavMs in CaCl_2_ 100 mM. (**a**,**b**) Potential of Mean force of Ca^2+^ as a function of the axial position in WT NavMS (**a**) and the mutant with charge Q*_f_* = −5*e* (**b**). (**c**,**d**) Average number of coordinating oxygens per calcium ion in axial bins with a thickness of 2.0 Å. (**c**) Wild-type NavMs; (**d**) NavMs mutant with Q*_f_* = −5*e*. The distance cutoff to identify both calcium-chloride and calcium-oxygen interactions was set to 3.5 Å. Color code is as follows. Blue line: number of coordinating water oxygens; green line: number of coordinating oxygens provided by aspartate and glutamates; red line; number of coordinating oxygens provided by other protein residues; black line: total number of coordinating oxygens; magenta line: number of coordinating chlorides. (**e**–**h**) Configuration of the selectivity filter of wild-type NavMS (**e**,**g**) and the mutant with charge Q*_f_* = −5*e* (**f**,**h**). All the structures correspond to the last frame of a 150 ns simulation in 0.10 M of CaCl_2_. Panels (**e**,**f**) show a side view of the SF; panels (**g**,**h**) show the top view. Glu178 is shown in red, while Asp179 is shown in purple. The backbone of Leu177 is shown in green. Calcium ions are portrayed as orange beads.

The position of the ion in the SF revealed by the Potential of Mean Force (PMF) shows that Ca^2+^ ions do visit the vestibule region of the WT channel, but they never enter into the SF (Figure 4a). The presence of an additional negative charge in the SF (S179D) is sufficient to pull in a Ca^2+^ ion that occupies a binding site centered at z = 6.5 Å (Figure 4b). The PMF minimum corresponding to this binding site has a depth of approximately 7.0 kcal/mol, which, at the simulation temperature of 300 K, corresponds to 11.5 *k_B_T*. The energy well is thus so deep that a single Ca^2+^ cannot leave the SF. Thus, to be consistent with the experimental recording of Ca^2+^ current (Figure 2e,g), calcium permeation must involve some sort of knock-on mechanism. The role of the aspartate residue in the SF is immediately highlighted by Figure 4e–h, which shows the configuration of the SF in the last frame of the simulation. The resident calcium ion appears to be directly bonded to D179 and to E178, both located on chain A (Figure 4). The interactions with the other glutamates of the SF are all water-mediated. In order to better characterize calcium hydration, in Figure 4c,d we plot the average number of coordinating oxygen atoms per calcium ion in axial bins with a thickness of 2.0 Å. Figure 4 shows that when a calcium ion enters into the SF, the number of hydrating water molecules drops from approximately 8 to 4.5. This dehydration is compensated by an increase in the number of coordinating oxygens provided by aspartate and glutamate residues (approximately 3). Thus, when Ca^2+^ enters the SF, the total number of coordinating oxygens remains roughly unchanged (Figure 4c,d).

A collective diffusion model approach was adopted to approximate the Ca^2+^ currents [46]. The algorithm relates the spontaneous permeation events at equilibrium with steady currents induced by small voltages. This approach thus enables the estimation of currents from equilibrium simulations; however, as it is based on linear response theory, its predictions are reliable only in a small voltage range. The results of the calculation are summarized in Table 2.

Notwithstanding the limitations of our calculations, the collective diffusion modelling predictions are in reasonable agreement with the experimental observations (Figure 2). For example, (1) whole-cell recordings showed that peak sodium currents increased by approximately 1.5-fold in the Q*_f_* = −5*e* NavMs channel (−35 to −55 pA/pF); this is mirrored by a 1.5-fold increase in sodium conductance (from 23.06 to 35.37 pS) predicted by linear response theory calculations. (2) Experimental measurements of peak calcium currents in the Q*_f_* = −5*e* mutant are approximately 10 times smaller than those for sodium. This is consistent with the modelling in the Q*_f_* = −5*e* mutant, in which a seven-fold greater sodium (35.37 pS) conductance is predicted compared to that for calcium (4.87 pS). (3) The small finite Ca^2+^ influx predicted in the WT NavMs (Table 2; 1.69 pS) can be observed in the electrophysiological recordings (Figure 2e).

## 4. Discussion and Conclusions

In this work, we engineered radial asymmetry in the bacterial NaChBac and NavMs channels as a first attempt to mimic the features of eukaryotic voltage-gated sodium and calcium channels. It is well known that prokaryotic sodium channels are characterized by a glutamate ring that imparts a charge −4*e* to the SF and endows the channel with Na^+^ selectivity. It is also well established that an increase in the negative charge of the SF makes the channel progressively more calcium-selective. Pioneering studies by the Clapham group, for instance, showed that mutating into aspartate either serine of the SF sequence TLESWAS of NaChBac decreases the P_Na_/P_Ca_ ratio, while a mutation of both serines makes the channel completely calcium-selective [20]. Using the same strategy, more recently Tang et al. replaced the TLESWSM sequence in the SF of NavAb with TLDDWSD, causing a complete shift from sodium to calcium selectivity [21]. It is noteworthy that, due to the tetrameric symmetry of prokaryotic NaVs, in all these studies the charge of the SF was always varied in −4*e* steps and radial symmetry was maintained. It is thus known that a charge Q*_f_* = −4*e* is typical of a Na+-selective channel, while a charge −8*e* or −12*e* leads to calcium selectivity. This change in the Q*_f_* value is rather coarse and does not address the fact that the SF of eukaryotic channels is asymmetric. Therefore, it is important to investigate the influence on the selectivity of charge changes by −1*e* steps.

The study of random heteroteramers in our work indicated that channels with an SF charge smaller than −4*e* mediate Na^+^ currents and channels with an SF charge in the −4*e* < Q*_f_* < −8e range conduct Ca^2+^. Furthermore, the study of the NavMs concatemer showed that the presence of an additional negative charge in the SF leads to a significant increase in the Na^+^ and Ca^2+^ current.

The electrophysiological behavior of the NaChBac populations of randomly assembled heterotetramers appears to be in reasonable agreement with the predictions of the Ionic Coulomb Blockade (ICB) model [23,24,25]. According to this model, ion permeation and selectivity through channels mainly depend on the Q*_f_* of the SF. If calcium permeation is plotted as a function of Q*_f_*, a pattern of alternating conductance and stop bands can be observed. In contrast, the same plot for sodium predicts a steep increase in current magnitude up to values in Q*_f_* of <−2*e*, followed by a plateau and the absence of stop bands (Figure 2 and Figure 3, in [25]), consistent with sodium permeation being relatively insensitive to changes in Q*_f_*. Thus, the predictions of the ICB model appear to be compatible with the plot of peak sodium currents in Figure 1c. Furthermore, it is tempting to envisage the pattern of Ca^2+^ current density shown in Figure 1d as an oscillation in the calcium conductance (i.e., conductance and stop bands), which would repeat over a wider range of Q*_f_* values. The ICB model, however, appears to be less successful in explaining the so-called ”EEEE paradox”—that is, the apparently shared “EEEE” motif in both the sodium-selective bacterial NaVs and the calcium-selective eukaryotic CaVs. Kaufman et al. tentatively reconciled this inconsistency, noting the presence of a conserved aspartate close to the EEEE ring of eukaryotic CaVs, thus redefining the motif as EEEED, which raises the SF charge to −5*e* [36]. Our experiments on the NavMs concatemer go some way towards confirming this prediction, but also highlight that other factors in addition to the value of Q*_f_* are important. The ICB model predicts that a charge −5*e* allows the access of a third Ca^2+^ ion when the SF is already occupied by two resident calcium ions. Our MD simulations, however, show that while no calcium ion gains access to the SF of WT NavMs, only a single Ca^2+^ ion stably occupies the filter of the mutant with charge −5*e*. This calcium ion is strongly bound to D179 and E178 located on the same subunit, and sits in a free energy well so deep that it cannot leave the SF. At the same time, the resident ion probably exerts an electrostatic repulsion on other potentially incoming Ca^2+^ ions, preventing a knock-on mechanism in a similar fashion as that described for NaChBac [49].

Our experiments and simulations thus suggest that the extra negative charge is effective in the capture of cations from the bulk, but it does not promote permeation. Contrary to that postulated by simplified physical models (in which the channel atomic structure is not considered), the charge of the SF is not the only determinant of conduction and selectivity. It is possible that calcium flow in eukaryotic Cavs requires some sort of fine modulation of charge effects. Flood et al., for instance, performed an interesting computational study grafting the SF and external vestibular region of the human NaV1.2 channel into the scaffold of the NavRh bacterial channel [35]. Their multi-microsecond MD simulations revealed that permeation and selectivity depend on the close interplay of the DEKA and EEDD rings, so that the charge of the extended filter region is −5*e* as in our NavMs mutant. In its protonated state, the lysine residue of the DEKA ring acts like a built-in sodium ion involved in the formation of multi-carboxylates/multi-ion complexes. When the charged ammonium group of lysine is in the HFS site, where the electrostatic potential is most negative, it creates a smooth electrostatic environment leading into the cavity, whereas, when it is bent toward the central cavity, it creates a zone of high electrostatic potential that cuts the cavity off from the SF. Our recent work [38], showing the possibility to create stable concatemers of the bacterial NavMs channel, offers the opportunity to experimentally test these computational predictions by creating a bacterial channel chimera where the SF and vestibule of the human Nav1.2 channel are grafted onto the NavMs concatemer.

Since no positively charged residue appears to be located close to the SF of eukaryotic CaVs, the fine modulation of the charge might rely on the differential protonation of the acidic residues of the EEEED locus. The effect of protonation has been extensively studied through MD simulations. Furini et al., for instance, showed that the glutamate side chains in NavAb can adopt two different orientations pointing either towards the extracellular environment or towards the central cavity [34]. Interestingly, they found that the likelihood of the inwardly directed arrangement increases when E177 residues are protonated. Moreover, the presence of a glutamate residue with the side chain directed to the central cavity increases the energy barrier for the translocation of sodium ions. Since E177 was observed to adopt an alternative conformation in MD simulations with Ca^2+^ ions [50], it is possible that these protonation-induced configurations also affect selectivity. While the control of the protonation state of the filter is a trivial task in MD simulations, it is a challenging endeavor in biophysical experiments.

This leads us to the methodological aspect of our work. Our study not only tested the importance of SF charge in controlling ion selectivity and permeation, but created new tools extending the use of bacterial channels as models of eukaryotic ones. Indeed, the current work is the first one to report experiments on a NaV channel in which the pore region has been mutated to have radial asymmetry, and thus it represents an important first step in bridging the major limitation in using bacterial sodium channels to investigate their eukaryotic counterparts. Our methodology will enable us to design physical experiments to investigate the mechanisms of the fine modulation of charge effects that are likely to occur in asymmetric eukaryotic channels, such as that predicted by Flood et al. [35].

A further methodological merit of our approach is its relevance in understanding the effect of pH on channel permeation and selectivity. In fact, when the pH is varied, the four glutamates of the SF are unlikely to be protonated or deprotonated simultaneously. A more probable scenario is that they are protonated or deprotonated one at a time, resulting in +1*e* or −1*e* changes in the SF charge [23]. Finally, our combination of molecular dynamics and electrophysiological approaches provided fresh insight into the molecular mechanisms of cation permeation in bacterial sodium channels, and gave insight into understanding the molecular mechanisms that underlie the function of NaVs and CaVs.

## Figures and Tables

**Table 1:** Probabilities of homo- and hetero-tetramer NaChBac channel formation in CHO cells co-transfected with 5 µg (total) of cDNAs encoding for wild-type (LESWAS) NaChBac and mutated NaChBac, in which the selectivity filter amino acid sequence was LASWAS or LEDWAS. Note that the Q*_f_* values for LASWAS, LESWAS, and LEDWAS monomers are 0, −1, and −2, respectively. Probabilities for channel formations were determined by Binomial distribution $P(n,N-n)=C(n,N-n)p^{n}{\left(1-p\right)}^{N-n}$.

cDNA Population Transfected into CHO Cells	LASWAS	LASWAS LESWAS (3:1)	LASWAS LESWAS (1:1)	LASWAS LESWAS (1:3)	LESWAS	LESWAS LEDWAS (3:1)	LESWAS LEDWAS (1:1)	LESWAS LEDWAS (1:3)	LEDWAS
Probabilities and Q*_f_* values for tetramer formation	Q*_f_* = 0: 100%	Q*_f_* = 0; 32% Q*_f_* = −1; 42% Q*_f_* = −2; 21% Q*_f_* = −3; 4% Q*_f_* = −4; 0.3%	Q*_f_* = 0; 6.2% Q*_f_* = −1; 25% Q*_f_* = −2; 37% Q*_f_* = −3; 25% Q*_f_* = −4; 6.2%	Q*_f_* = 0; 0.3% Q*_f_* = −1; 4% Q*_f_* = −2; 21% Q*_f_* = −3; 42% Q*_f_* = −4; 32%	Q*_f_* = −4; 100%	Q*_f_* = −4; 32% Q*_f_* = −5; 42% Q*_f_* = −6; 21% Q*_f_* = −7; 4% Q*_f_* = −8; 0.3%	Q*_f_* = −4; 6.2% Q*_f_* = −5; 25% Q*_f_* = −6; 37% Q*_f_* = −7; 25% Q*_f_* = −8; 6.2%	Q*_f_* = −4; 0.3% Q*_f_* = −5; 4% Q*_f_* = −6; 21% Q*_f_* = −7; 42% Q*_f_* = −8; 32%	Q*_f_* = −8; 100%

**Table 2:** Current estimates through linear response theory. The first column shows the NavMs species analyzed, either the wild-type form EEEE with an SF charge Q*_f_* = −4*e* or the mutant EEEED with an additional negative charge in the SF (Q*_f_* = −5*e*). The second column shows the ion carrying the current, the third column reports the estimated conductance in pS, and the fourth column lists the estimated current at V = −20 mV. This voltage corresponds to the peak current in the current–voltage plots determined from whole-cell patch-clamp experiments.

Species	Ion	Conductance (pS)	Currents (pA; −20 mV)
EEEE	Ca^2+^	1.69	−0.033
EEEED	Ca^2+^	4.87	−0.097
EEEE	Na^+^	23.06	−0.46
EEEED	Na^+^	35.37	−0.70

## Data Availability

Data related to this research are openly available from the University of Warwick archive at (https://wrap.warwick.ac.uk/143573). Fedorenko, Olena A., Khovanov, Igor A., Roberts, Stephen K., and Guardiani, Carlo (2020) Data for *Changes in ion selectivity following asymmetrical addition of charge to the selectivity filter of bacterial sodium channels* [Dataset].

## Supplementary Materials

The following are available online at https://www.mdpi.com/1099-4300/22/12/1390/s1: Supplementary Table S1 Parameters of NavMs equilibration. Supplementary Table S2. Peak currents ratios of WT and mutant NavMs channels. Supplementary Table S3. Peak currents ratios of NaChBac heterotetramers. Supplementary Figure S1. The tetramer formation via co-transection is proved to be a random process without bias for homo- or hetero-tetramer formation. Supplementary Figure S2. The single channel currents recorded from WT NaChBac. Supplementary Figure S3. Schematic representation of NavMs concatemer. Supplementary Figure S4. Ion occupancy of Selectivity Filter in MD simulations of WT NavMs and its mutant with Q*_f_* = −5*e* in NaCl 140 mM. Supplementary Figure S5. Ion occupancy in Selectivity Filter in MD simulations of WT NavMs and its mutant with Q*_f_* = −5*e* in CaCl_2_ 100 mM. Supplementary Figure S6. Current-voltage plot calculations: comparison of constant electric field simulations and equilibrium simulations in conjunction with linear response theory.
