# Supplementary material for: Changes in Ion Selectivity Following the Asymmetrical Addition of Charge to the Selectivity Filter of Bacterial Sodium Channels
Source: Entropy (Basel). 2020 Dec 9;22(12):1390. doi: 10.3390/e22121390 (PMC7764494; doi:10.3390/e22121390)
Supplement: Supplementary file 1 [file entropy-22-01390-s001.pdf]

Supplementary table 1. Parameters of NavMs equilibration. For each stage the force constants are listed of the harmonic restrants on backbone (BB), side-chains (SC), phospholipid heads (Lip-Head), and dihedral angles (Dihed). Force constants are expressed in kcal/mol/Å2. Also shown is the time-step δt (in fs) and the duration Tsim (in ps) of each stage. This protocol was applied only to protein sequence 155-193 while the backbone of the trans-membrane helices (residues 131-154 and 194-222) was harmonically restrained with a force constant of 50 kcal/mol/Å2 for the whole duration of the simulation

	stage-1	stage-2	stage-3	stage-4	stage-5	stage-6
BB	10.0	5.0	2.5	1.0	0.5	0.1
SC	5.0	2.5	1.0	0.5	0.1	0.0
Lip-Head	5.0	5.0	2.0	1.0	0.2	0.0
Dihed	500.0	200.0	100.0	100.0	50.0	0.0
δt	1.0	1.0	1.0	2.0	2.0	2.0
T_{sim}	25.0	25.0	25.0	200.0	200.0	200.0

Supplementary table 2. Peak currents ratios of WT and mutant NavMs channels.

Form	I_{peak}^{Na} (pA/pF)	I_{peak}^{Ca} (pA/pF)	$I_{peak}^{Ca}/I_{peak}^{Na}$
WT	41.66	0.75	0.018
DI	58.30	4.95	0.080
DII	58.30	5.70	0.097

Supplementary table 3. Peak currents ratios of NaChBac heterotetramers.

Species	I_{peak}^{Na} (pA/pF)	I_{peak}^{Ca} (pA/pF)	$I_{peak}^{Ca}/I_{peak}^{Na}$
LASWAS	0.0	0.0	0.0
3LAS:1LES	7.27	0.0	0.0
1LAS:1LES	21.82	0.0	0.0
1LAS:3LES	50.90	0.66	0.013
LESWAS	65.45	0.66	0.010
3LES:1LED	109.09	6.0	0.054
1LES:1LED	65.45	16.66	0.25
1LES:3LED	76.36	16.0	0.21
LEDWAS	72.72	8.0	0.11

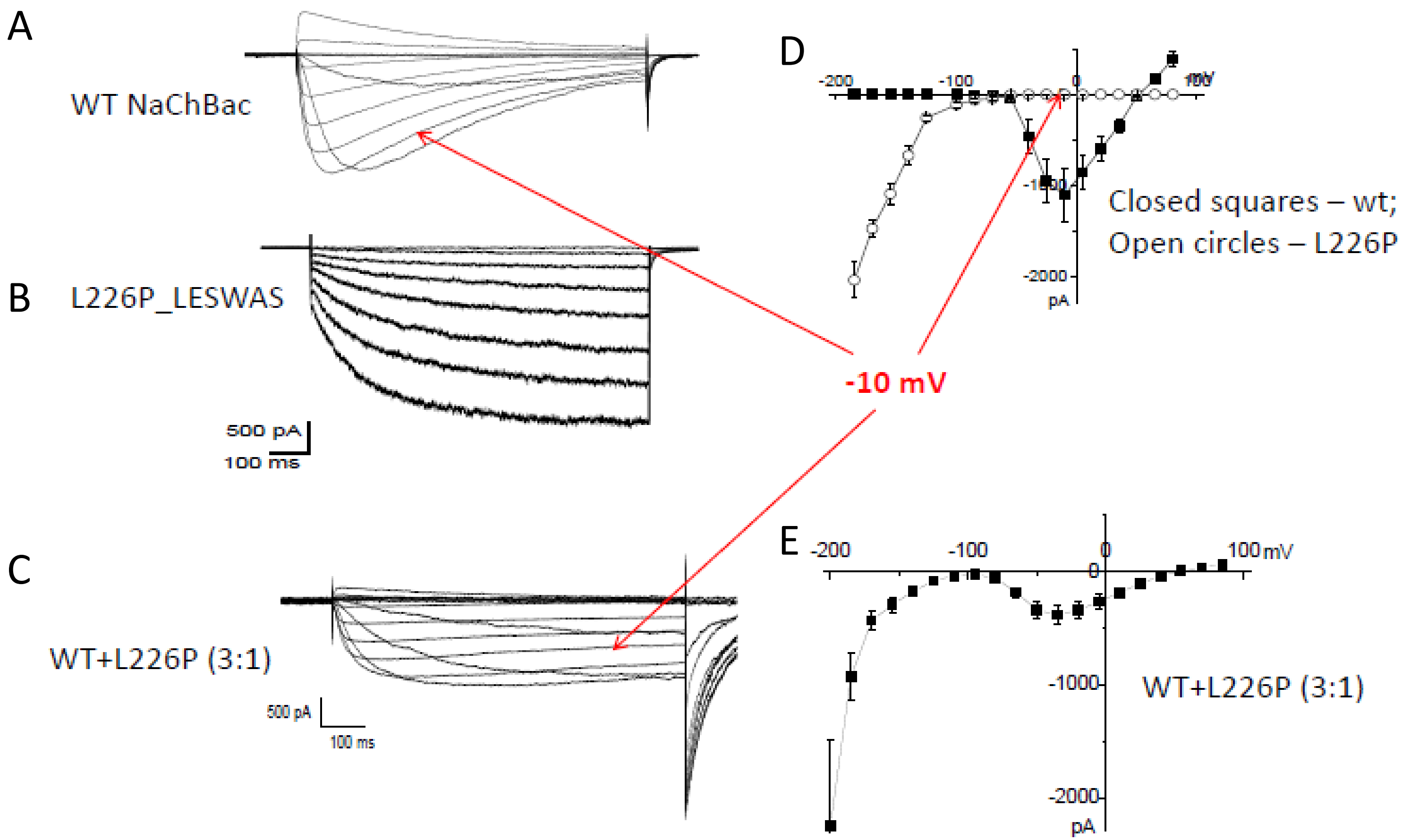

Supplementary Figure 1. The tetramer formation via co-transection is proved to be a random process without bias for homo- or hetero-tetramer formation. L226P_LESWAS mutant was employed to verify unbiased heterotetramer formation from NaChBac monomers exhibiting different amino acid sequences. The original recordings of the whole cell currents mediated by wt NaChBac (A), L226P mutant channels (B) and 3:1 WT:L226P ratio transfection (C), and the current voltage relationships of these current (D and E).

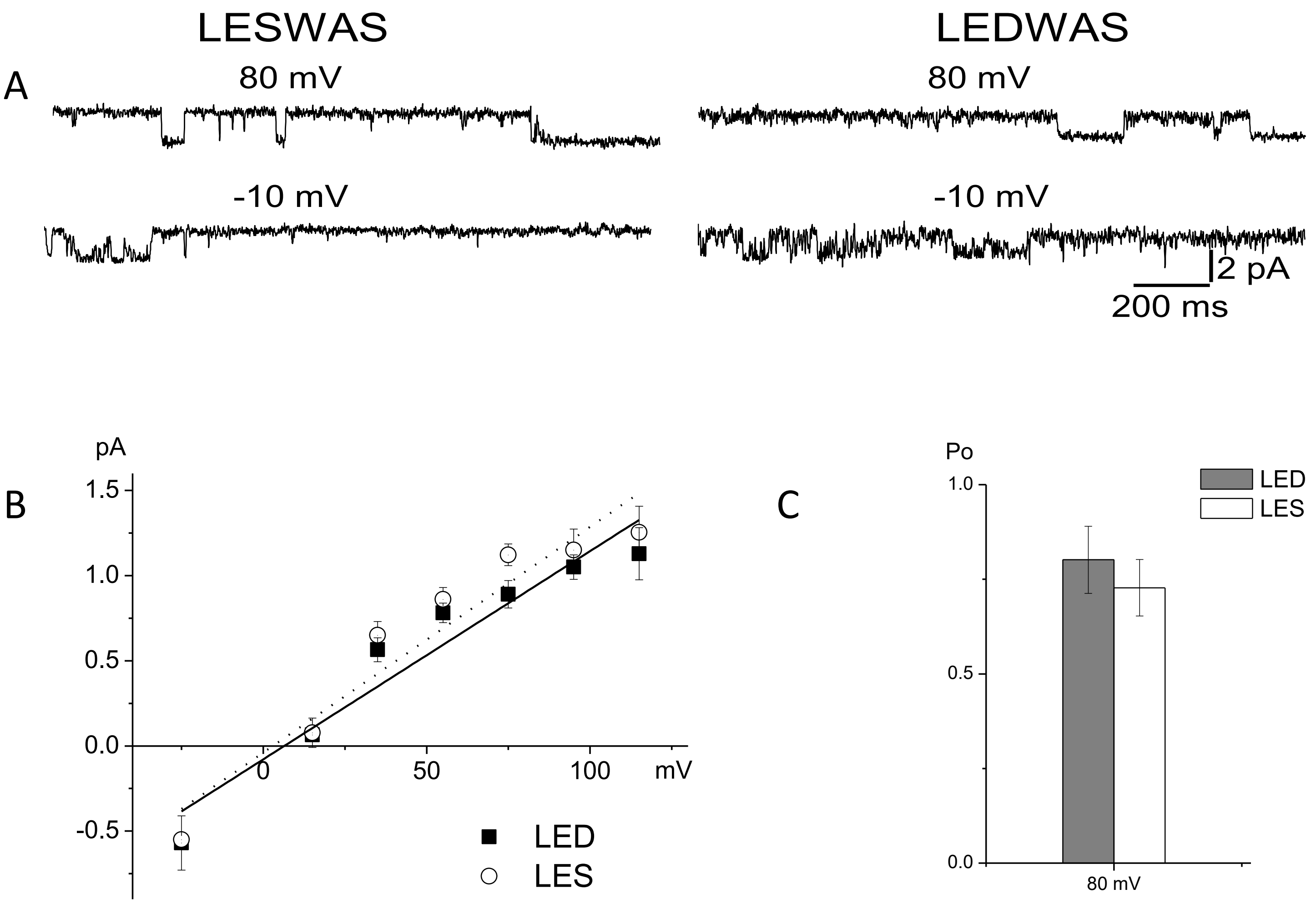

Supplementary Figure 2. The single channel currents recorded from wtNaChBac (LESWAS; left trace) and LEDWAS (right trace) mutant at 80 mV or at -10 mV (A), current-voltage relationships (B; open circles – LESWAS, closed squares - LEDWAS) and open probability plot (C), built using such recordings (n=3-4) revealed no dramatic changes in channels' activity due to the point mutation in its selectivity filter.

A

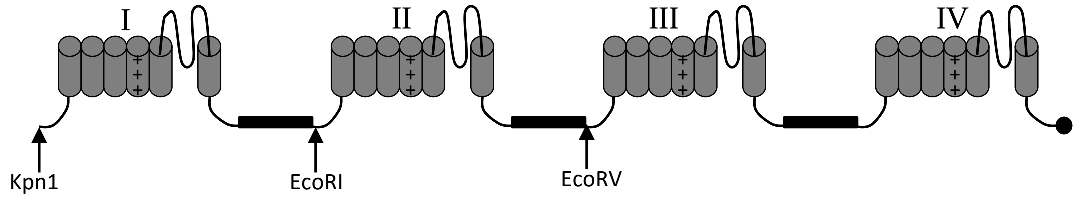

B

NavMs – WT (Q*f*=-4*e*)

domain I T L **E** S W S M
domain II T L **E** S W S M
domain III T L **E** S W S M
domain IVT T L **E** S W S M

NavMs – DII mutant (Q*f*=-5*e*)

domain I T L **E** S W S M
domain II T L **E** **D** W S M
domain III T L **E** S W S M
domain IVT T L **E** S W S M

C

CaV1.2

domain I F Q C I T M **E** G W T D
domain II F Q I L T G **E** **D** W N S
domain III F T V S T F **E** G W P E
domain IV F R C A T G **E** A W Q D

Supplementary Figure 3. Schematic representation of NavMs concatemer. (A) Each domain corresponding to monomeric NavMS is shown as composed of six transmembrane spanning (TMS) domains (the '+' denote voltage sensing TMS S4) and the pore region between TMS 5 and 6. Arrows denote position of unique DNA restriction sites engineered into the cDNA constructs and used to generate different mutants (see Methods). The SF region amino acid sequence is shown in red squares (B); for WT NavMS four glutamates in the EEEE ring give Q*f*=-4*e*; adding aspartate (D, in red) in one domain (for example domain II) leads to formation of Q*f*=-5*e*, which resembles to the sequences in native CaV1.2 channel (C).

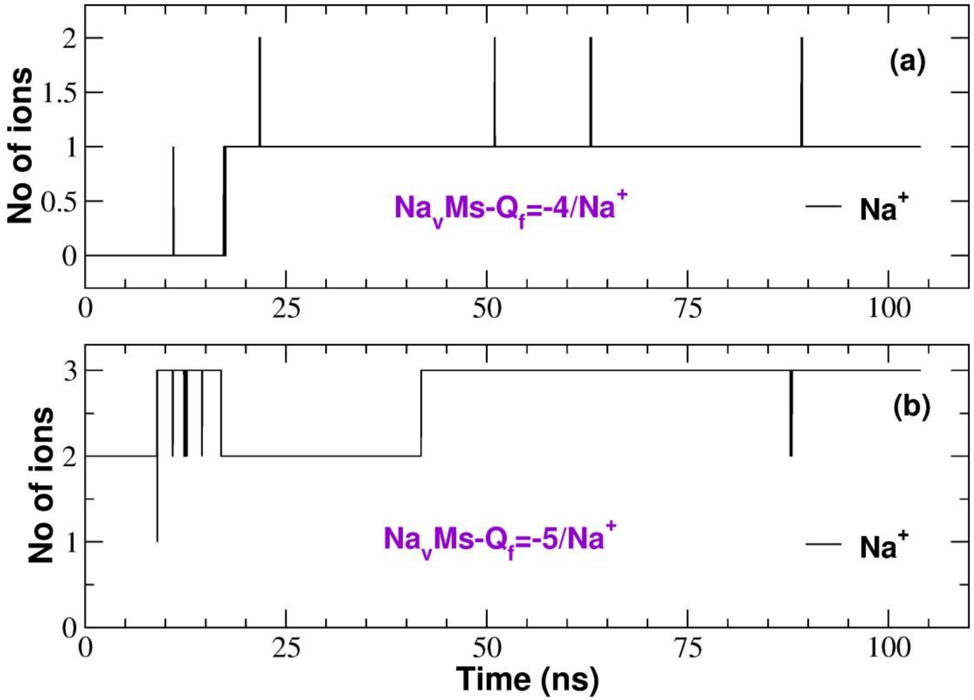

Supplementary Figure 4. MD simulations of WT NavMs and its mutant with Q*f*=-5*e* in NaCl 140 mM. Time evolution of the number of sodium ions occupying the SF of WT NavMS (A) and the mutant with charge Q*f*=-5*e* (B). The SF is defined as the axial 5 region [0 : 13] Å.

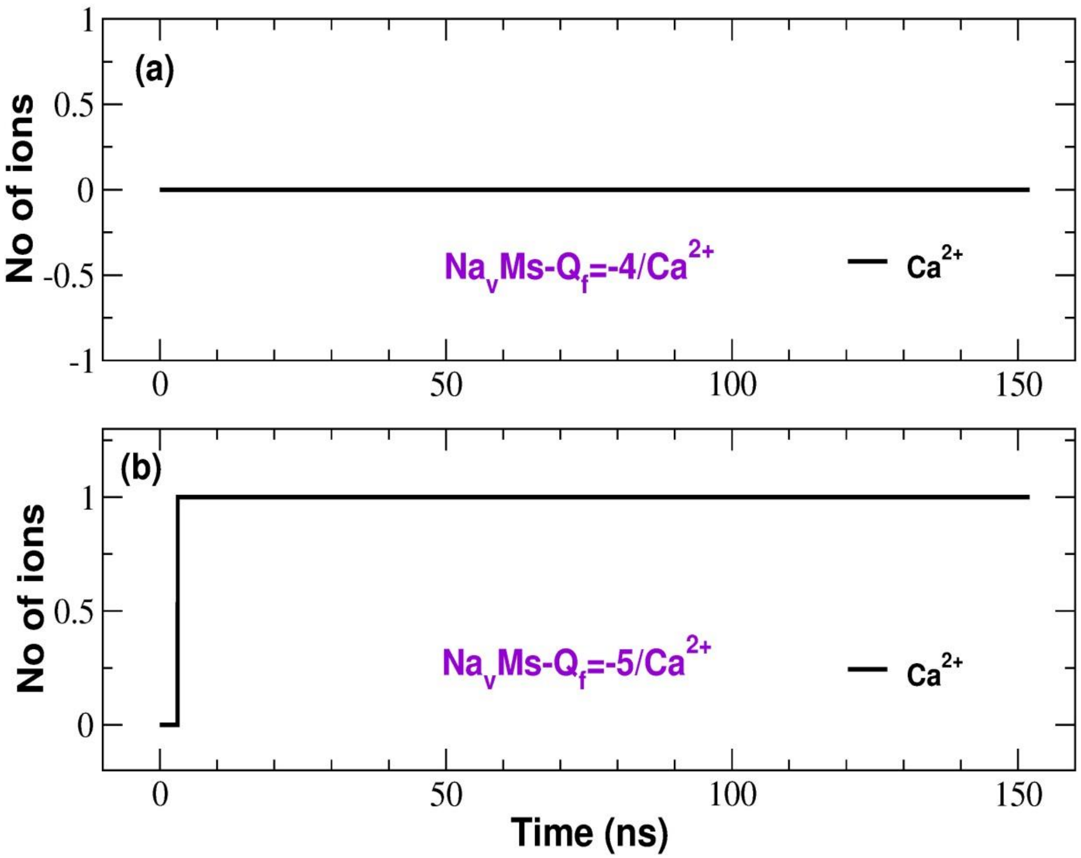

Supplementary Figure 5. MD simulations of WT NavMs and its mutant with Q*f*=-5*e* in $CaCl_2$ 100 mM. Time evolution of the number of calcium ions occupying the SF of WT NavMS (A) and the mutant with charge Q*f*=-5*e* (B). The SF is defined as the axial region [0 : 13] Å.

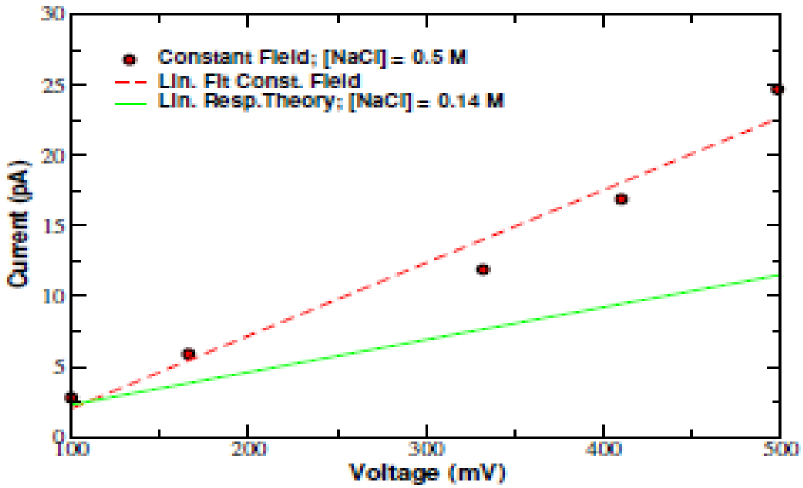

Supplementary Figure 6. Current-voltage plot calculations: comparison of constant electric field simulations and equilibrium simulations in conjunction with linear response theory. The red dots show the current values computed by Ulmschneider et al [1] at a few different voltages; the red dashed line is a linear fit interpolating the current points. The green solid line is the current-voltage plot computed in our work using equilibrium simulations and processing the trajectories with the aid of linear response theory. The different slope of the two lines and thus the different conductance reflects the different NaCl concentrations.

The NavMs channel has been extensively characterized by Ulmschneider et al. [1] with microsecond scale simulations with a constant external electric field. The current-voltage plot derived by Ulmschneider et al, however, cannot be quantitatively compared to our results because, besides a different force field and a different computational technique, in order to enhance the number of permeation events, these authors used a higher NaCl concentration (0.5 M vs 0.14 M in our simulations) and a higher temperature (310 K vs 300 K). The higher sodium conductance reported by Ulmschneider (40 pS) is approximately twice the one computed from our simulation (23 pS) and roughly accounts for the difference in salt concentration. A comparison of the current-voltage plots computed in [Ulmschneider et al, 2013] and by us is shown below.

Reference:

[1] Ulmschneider, M.B., C. Bagneris, E.C. McCusker, P.G. DeCaen, M. Delling, D.E. Clapham, J.P. Ulmschneider, and B.A. Wallace. 2013. Molecular dynamics of ion transport through the open conformation of a bacterial voltage-gated sodium channel. Proc. Natl. Acad. Sci. USA 110(16):6364-6369.
